# Supplementary material for: Age-Related Changes in Lipid and Glucose Levels Associated with Drug Use and Mortality: An Observational Study
Source: J Pers Med. 2022 Feb 14;12(2):280. doi: 10.3390/jpm12020280 (PMC8876997; doi:10.3390/jpm12020280)
Supplement: Supplementary file 1 [file jpm-12-00280-s001.zip › jpm-1547850-supplementary.pdf]

Supporting information for: Age-related Changes in Lipid and Glucose Levels Associated with Drug Use and Mortality: An Observational Study

Rene Markovič [1, 2] Vladimir Grubelnik [2], Helena Blažun Vošner [3, 4, 5], Peter Kokol [2], Matej Završnik [6], Karmen Janša [7], Marjeta Zupet [7], Jernej Završnik [3, 5, 8, 9*] and Marko Marhl [1, 10, 11*]

1 Faculty of Natural Sciences and Mathematics, University of Maribor, SI-2000 Maribor, Slovenia
2 Faculty of Electrical Engineering and Computer Science, University of Maribor, SI-2000 Maribor, Slovenia
3 Community Healthcare Center Dr. Adolf Drolc Maribor, SI-2000 Maribor, Slovenia
4 Faculty of Health and social Sciences, SI – 2380 Slovenj Gradec, Slovenia
5 Alma Mater Europaea – ECM, SI – 2000 Maribor, Slovenia
6 University Medical Center Maribor, Ljubljanska ulica 5, SI-2000 Maribor, Slovenia
7 The Health Insurance Institute of Slovenia, Miklošičeva cesta 24, SI-1507 Ljubljana, Slovenia
8 Faculty of Natural sciences and Mathematics, University of Maribor, SI- 2000 Maribor, Slovenia
9 Science and Research Center Koper, SI- 6000 Koper, Slovenia
10 Faculty of Education, University of Maribor, SI-2000 Maribor, Slovenia
11 Faculty of Medicine, University of Maribor, SI-2000 Maribor, Slovenia
*Correspondence: M.M.: marko.marhl@um.si, J.Z.: jernej.zavrsnik@zd-mb.si

Table of content

The initial statistical description of the CHDAD and HIIS dataset 2
Serum Cholesterol values 3
Serum LDL and HDL values 6
Serum Triglycerides values 8
Serum Glucose values 9
Statistical description of serum Gluc, Chol, LDL, HDL, and TG levels 12
Drug usage analysis 13
Diagnostic criteria for T2DM 14
List of DFD and LMA drugs considered 15

The initial statistical description of the CHDAD and HIIS dataset

Here is a basic statistical description of the CHDAD and HIIS datasets. Table S1 describes the age- and sex-specific characteristics of CHDAD, which is related to the laboratory test results. More specifically, Table S1 provides information on the number of males, females, and all patients. In addition, Table S1 also reports the average number of test results per patient, which is reported in parentheses. Finally, we also report the ratio of female to male patients. We find that the prevalence of female patients is more pronounced in the younger (under 40) and older (over 70) age groups. The ratio is lowest in the age groups between 40 and 70 years, corresponding to a more balanced ratio between women and men. As for the number of patients, it is highest in the 60-69 age group. The number of tests per patient also varies. In general, we observe an increasing number of tests per patient until the age group 70-79. The average total number of tests per patient is 2.1. Due to this fact, in our analyses where person-specific values are needed, we calculate the mean of all reported test results for a person when more than one test result is reported. We also take age group into account. Thus, only an individual's test scores obtained while the individual was assigned to an age group are averaged. The test results of the same person who belongs to a different age group are analyzed accordingly within the other age group.

Table S1: CHDAD dataset description. Based on the laboratory test and age group, the table gives the number of individual male, female, and all patients within a given age group. In addition to the number of patients, we also report the average number of test results per patient in parentheses. Finally, the ratio between female and male patients is given.

	Age group							
Gluc	**20-29**	**30-39**	**40-49**	**50-59**	**60-69**	**70-79**	**80-89**	**90+**
Male	231 (1.2)	911 (1.4)	1878 (1.7)	2497 (2.3)	3066 (2.9)	2694 (3.4)	1635 (3.4)	489 (2.7)
Female	444 (1.2)	1517 (1.5)	2269 (1.8)	2888 (2.3)	3322 (3.0)	3121 (3.5)	2184 (3.5)	1159 (2.7)
Both	675 (1.2)	2428 (1.4)	4147 (1.8)	5385 (2.3)	6388 (2.9)	5815 (3.5)	3819 (3.5)	1648 (2.7)
F/M	1.92	1.67	1.21	1.16	1.08	1.16	1.34	2.37
Chol								
Male	113 (1.3)	693 (1.3)	1776 (1.7)	2413 (2.1)	2978 (2.7)	2603 (3.1)	1513 (2.8)	376 (2.2)
Female	172 (1.2)	835 (1.3)	2001 (1.6)	2755 (2.1)	3250 (2.8)	3028 (3.3)	2045 (2.9)	853 (2.3)
Both	285 (1.2)	1528 (1.3)	3777 (1.6)	5168 (2.1)	6228 (2.8)	5631 (3.2)	3558 (2.9)	1229 (2.2)
F/M	1.52	1.20	1.13	1.14	1.09	1.16	1.35	2.27
LDL								
Male	98 (1.3)	624 (1.3)	1675 (1.6)	2325 (2.1)	2942 (2.6)	2565 (3.0)	1475 (2.7)	340 (2.2)
Female	149 (1.1)	713 (1.3)	1815 (1.6)	2639 (2.0)	3186 (2.7)	2986 (3.2)	1999 (2.8)	778 (2.2)
Both	247 (1.2)	1337 (1.3)	3490 (1.6)	4964 (2.1)	6128 (2.7)	5551 (3.1)	3474 (2.8)	1118 (2.2)
F/M	1.52	1.14	1.08	1.14	1.08	1.16	1.36	2.29
HDL								
Male	97 (1.3)	622 (1.3)	1676 (1.6)	2321 (2.1)	2946 (2.6)	2568 (3.0)	1476 (2.7)	339 (2.2)
Female	150 (1.1)	714 (1.3)	1818 (1.6)	2643 (2.0)	3188 (2.7)	2985 (3.1)	1996 (2.8)	772 (2.2)
Both	247 (1.2)	1336 (1.3)	3494 (1.6)	4964 (2.1)	6134 (2.7)	5553 (3.1)	3472 (2.8)	1111 (2.2)
F/M	1.55	1.15	1.08	1.14	1.08	1.16	1.35	2.28

TG								
Male	107 (1.3)	663 (1.3)	1721 (1.7)	2369 (2.1)	2972 (2.6)	2589 (3.0)	1505 (2.7)	360 (2.2)
Female	163 (1.2)	793 (1.3)	1924 (1.6)	2704 (2.1)	3236 (2.8)	3013 (3.2)	2030 (2.9)	823 (2.2)
Both	270 (1.2)	1456 (1.3)	3645 (1.6)	5073 (2.1)	6208 (2.7)	5602 (3.1)	3535 (2.8)	1183 (2.2)
F/M	1.20	1.12	1.14	1.09	1.16	1.35	2.29	1.52

See Table S2 for the HIIS data set's description of medication use. We can see that regardless of the type of medication, the highest number of patients is found in the age group 60-69. For the drugs used for diabetes, we observe female prevalence up to age group 30-39 and from age group 70-79. The prevalence of male patients for this type of drug is observed in age groups 40-69. For lipid-modifying agents, the prevalence of male patients is observed till the age group 60-69. From this age group onwards, female patients outnumber male patients for lipid-modifying drugs.

Table S2: HIIS dataset description. Given are the number of individual male, female, and all patients within a given age group. In addition, the number of all, male and female patients is also given for drugs used for diabetes and lipidmodifying agents. We also report the ratio of females to males (F/M). The last column (Total) represents the sum of the corresponding rows and the ratio of female to male patients. It should be noted that the total row does not correspond to the number of individual patients, as a patient may be found in more than one age group.

	Age group							
**Drugs used in diabetes**	20-29	30-39	40-49	50-59	60-69	70-79	80-89	90+
Male	2,276	4,305	4,401	12,266	22,471	25,338	17,787	3,462
Female	680	2,035	7,340	21,952	33,035	24,007	9,984	1,001
Both	2,956	6,340	11,741	34,218	55,506	49,345	27,771	4,463
F/M	3.35	2.12	0.60	0.56	0.68	1.06	1.78	3.46
**Lipid modifying agents**								
Male	249	1,314	7,471	38,052	68,662	66,371	39,829	5,762
Female	382	3,553	17,301	52,331	76,411	55,001	22,984	2,112
Both	631	4,867	24,772	90,383	145,073	121,372	62,813	7,874
F/M	0.65	0.37	0.43	0.73	0.90	1.21	1.73	2.73
**Both drug types**								
Male	2,478	5,294	10,076	42,094	74,358	72,580	46,055	7,628
Female	997	4,953	20,672	59,477	84879	60,954	26,091	2,574
Both	3,475	10,247	30,748	101,571	159,237	133,534	72,146	10,202
F/M	2.49	1.07	0.49	0.71	0.88	1.19	1.77	2.96

Serum Cholesterol values

The CHDAD dataset was first examined for the distribution of serum cholesterol levels in different age groups. Figure S1 shows that the number of test samples generally increases with age (dashed blue line), peaking in patients who are 65 years old. The mean serum cholesterol values exceed the upper limit in patients older than 30 years (horizontal red line). Mean and median values exceed the upper limit in patients older than 35 years. From this age on, the mean and median values are permanently above the upper limit for normal serum cholesterol levels. The highest mean and median values for cholesterol are found in patients who are 53 years old. Although the number of

tests still increases after age 53, the mean and median values begin to decrease. The most common serum cholesterol value (mode) for patients in the age range of 48 to 62 years is above the upper limit for normal cholesterol levels. For patients below or above this age range, the mode value of serum cholesterol levels fluctuates between normal and elevated cholesterol levels.

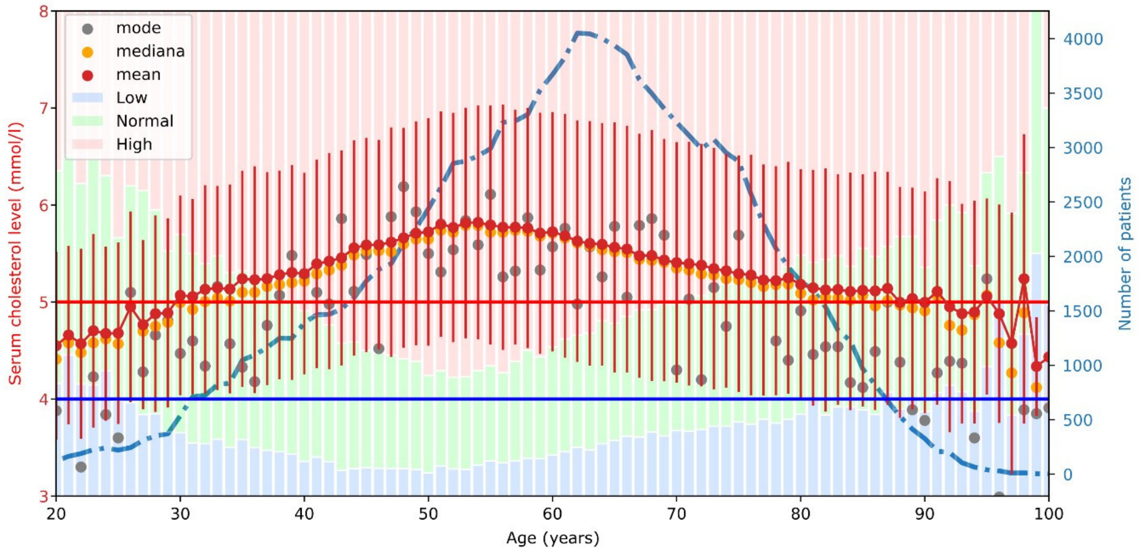

Figure S1: Age-specific results of blood cholesterol levels. The dashed blue line shows the absolute number of tests performed for a given age group. Gray, orange, and red circles indicate the mode, median, and mean blood cholesterol levels for a given age group. Vertical red lines indicate the standard deviation of blood cholesterol levels within a given age group. Horizontal red line marks the upper normal blood cholesterol level, and the horizontal blue line marks the lower normal blood cholesterol level. Blue, green and red bars highlight the relative contribution of test results, with low, normal, and high cholesterol levels, respectively.

The dynamics of serum cholesterol levels are gender-specific. Serum cholesterol levels in men and women in different age groups are shown in Figure S2. In general, the distribution of serum cholesterol levels in a given age group differs significantly between males and females. Male patients aged 30 to 50 years have higher serum cholesterol levels than female patients. The dispersion of serum cholesterol levels is higher in male patients aged 30 to 60 years. Among patients above 60, female patients have higher mean serum cholesterol levels and similar value dispersion as male patients. The proportion of patients with serum cholesterol above normal also reveals gender differences (Figure S2). The highest proportion of patients with abnormal serum cholesterol levels is found in male patients in the age group 45 - 50, while for female patients, the highest proportion is found in the age group 55 - 60. In addition, we also observe that the share of female patients with elevated serum cholesterol levels is higher when compared to male patients.

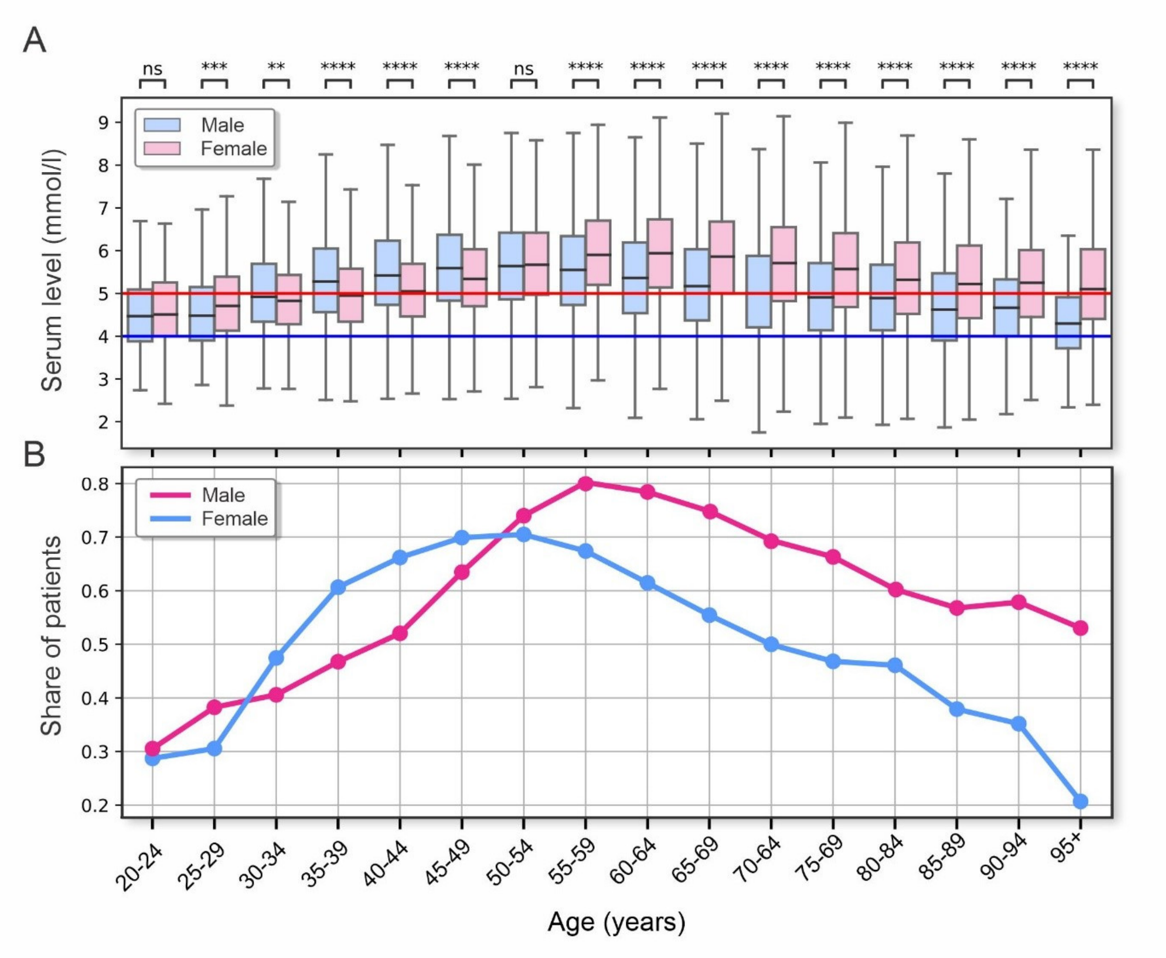

Figure S2: Age and sex in relation to serum cholesterol level. (A) Distribution of serum cholesterol levels for different age groups and sexes. (B) The proportion of patients with elevated serum cholesterol levels in different age groups and sexes. Asterix symbols in panel A indicate the level of statistical significance ($p < 0.0001$ - ****; $p < 0.005$ - ***, $p < 0.01$ - **; $p < 0.05$ - *; ns - not significant). Statistical significance was calculated using the Mann-Whitney U test.

Serum LDL and HDL values

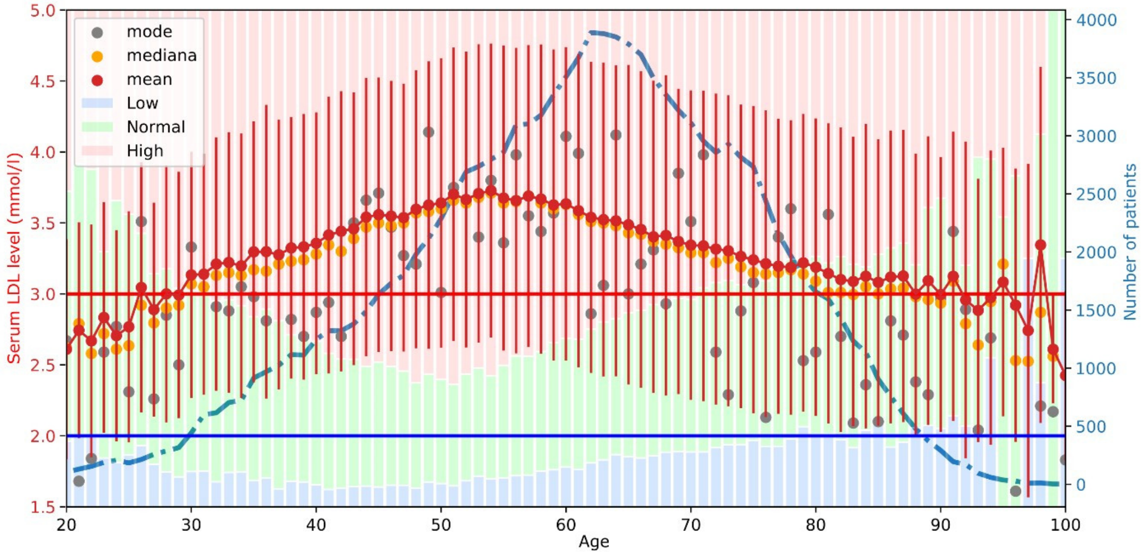

Figure S3: Age-specific results of LDL blood levels. The dashed blue line shows the absolute number of tests performed for a given age group. Gray, orange, and red circles indicate the mode, median, and mean blood LDL levels for a given age group. Vertical red lines indicate the standard deviation of blood LDL levels within a given age group. Horizontal red line marks the upper normal LDL level, and the horizontal blue line marks the lower normal blood LDL level. Blue, green, and red bars mark the proportion of test results with low, normal, or high LDL levels.

Overall, the results presented in Figure S3 for serum LDL levels show a high degree of similarity to the results obtained for serum cholesterol levels (see Figure S1). A higher proportion of serum LDL tests obtained from patients older than 30 years is above normal. The mean and median values are above the upper limit for tests obtained from patients older than 43 years. The mean serum LDL level increases until age 53 when it peaks. The most common serum LDL level (mode) in the age range of 43 to 60 is above the upper limit for serum LDL levels. The mode value of serum LDL levels oscillates between normal and elevated cholesterol levels for test results below or above this age range. The dynamics of serum LDL values are gender-specific. The dispersion of serum LDL values in men and women in different age groups is shown in Figure S4. The distribution of the measured serum LDL values for the first two age groups (20-24 and 25-29) shows no significant differences between male and female patients. No significant difference is also observed for test results obtained for male and female patients aged 50 till 54. Test results from male patients younger than 50 years have a higher mean and greater dispersion than female test results. From this age, 54 years, test results obtained from female patients have a higher mean and greater dispersion.

The proportion of male test results with above normal serum LDL levels initially increases more rapidly than the proportion of female test results, peaking in the 45-49 age group. After that, this proportion begins to decline in male test results. The proportion of female test results with above normal serum LDL levels continues to increase and exceed males' proportion. Among female patients, the proportion of above normal test results peaks in the 55- to 59-year-old age group.

The test results for serum HDL level show a significant difference between male and female patients. In general, the mean value of the test results is lower in male patients than in female patients in any given age group. Moreover, the calculated mean of test results in males is lower than the recommended serum HDL level. The mean of test results of female patients is generally higher than the recommended baseline serum HDL level. Therefore, the proportion of male test results that are lower than the recommended baseline level is continuously much higher than the proportion of female test results.

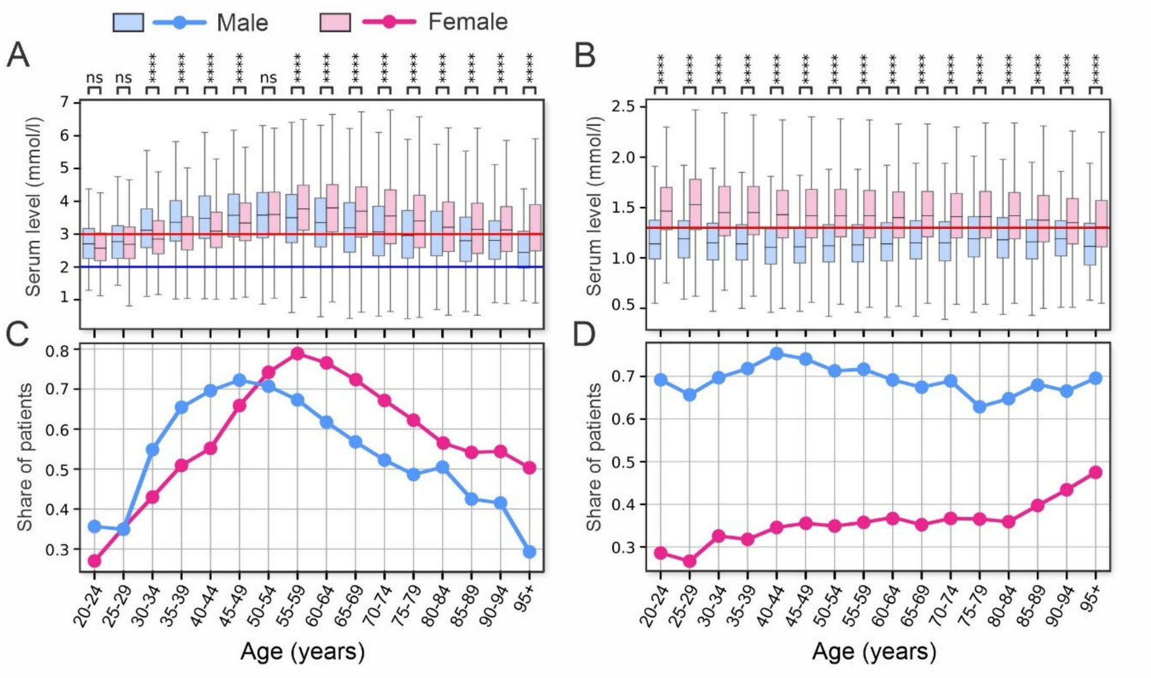

Figure S4: Age- and sex-specific serum LDL and HDL levels. (A) and (B) Distribution of serum LDL and HDL levels for different age groups and sexes, respectively. (C) and (D) Proportion of patients with abnormal serum LDL and HDL levels for different age groups and sexes, respectively. Asterix symbols in panels A and B indicate the level of statistical significance (p < 0.0001 - ****; p < 0.005 - ***, p < 0.01 - **; p < 0.05 - *; ns - not significant). Statistical significance was calculated using the Mann-Whitney U test.

Serum Triglycerides values

Figure S5 shows that the mean serum triglycerides (TG) hardly exceed the upper limit for normal serum TG values. However, the mean and mode values are always within the normal serum TG range. Mean TG values are elevated in test results of patients between 47 and 61 years of age. Before and after this age range, mean serum TG are normal.

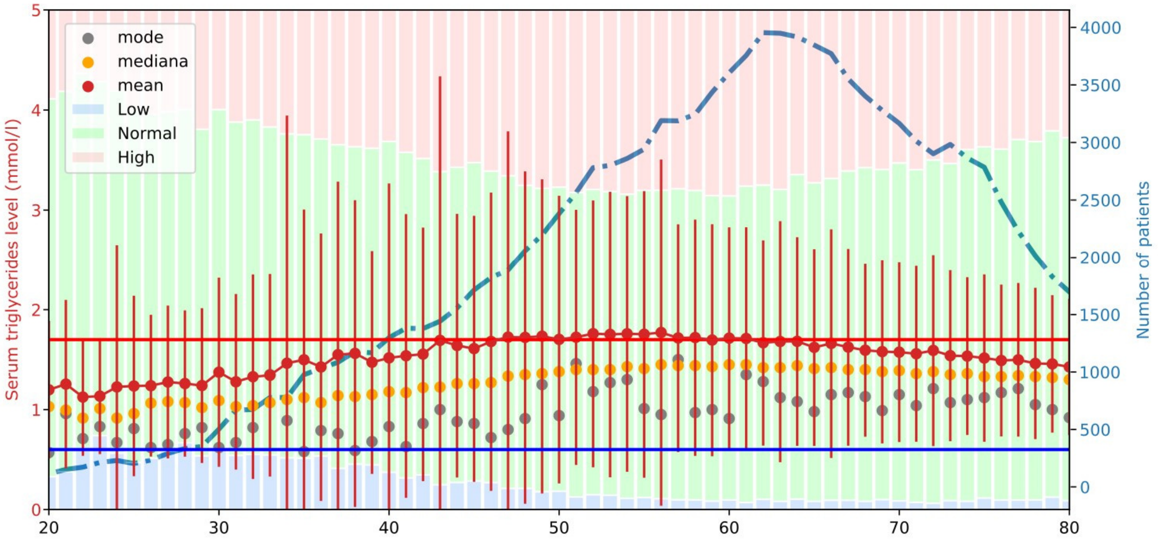

Figure S5: Age-specific results of blood triglyceride levels. The dashed blue line shows the absolute number of tests performed in patients of a given age. Gray, orange, and red circles indicate the mode, median, and mean blood triglyceride levels, respectively. Vertical red lines indicate the standard deviation of blood triglyceride levels around the mean values. Horizontal red and blue lines mark the range of normal serum levels TG.

Our analysis of TG levels shows (see Figure 6A) a significant difference between male and female patients in all age groups, except in the youngest age group, 20-24. In general, the mean value of test results is higher in male patients than in female patients up to the age group 75-79. From this age group onwards, the test results obtained from female patients have a higher mean value. We also observe that the dispersion of TG values is higher in male test results from age group 25-29 to age group 75-79. Regarding the proportion of elevated TG tests (see Figure 6B), we observe that the proportion is larger for males than for females up to the 75-79 age group. The peak in the proportion of above-normal test results is reached in males in the 50-54 age group and in females in the 65-69 age group 65-69.

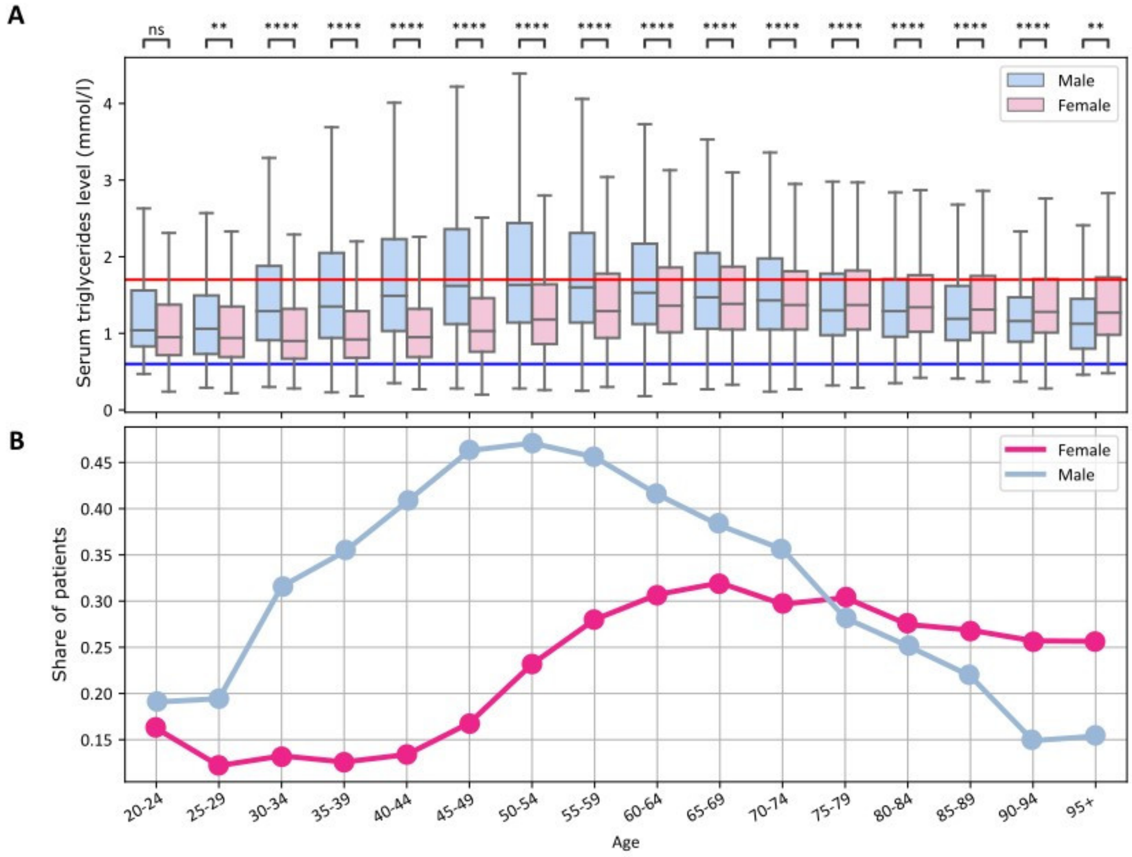

Figure S6: Age- and sex-specific serum triglyceride levels. (A) Distribution of serum triglyceride levels for different age groups and sexes. (B) The proportion of patients with elevated serum triglyceride levels for different age groups and sexes.

Serum Glucose values

The results, which focus on the measurement of serum glucose levels, are shown in Figure S7. The number of patients increases up to the age of 65 years. The mean serum glucose level increases until the age of 75 years and then starts to decrease. The mean serum glucose level exceeds the upper normal limit (horizontal red line) in the test results of patients older than 51 years. However, the median value never exceeds the upper limit for normal serum glucose levels. Median and mean serum glucose levels diverge with age, indicating a distribution with increasing positive skewness. This contrasts with the distribution of serum LDL and serum cholesterol levels, where the median and mean converge, indicating a decreasing skewness of the distribution and a more normal distribution.

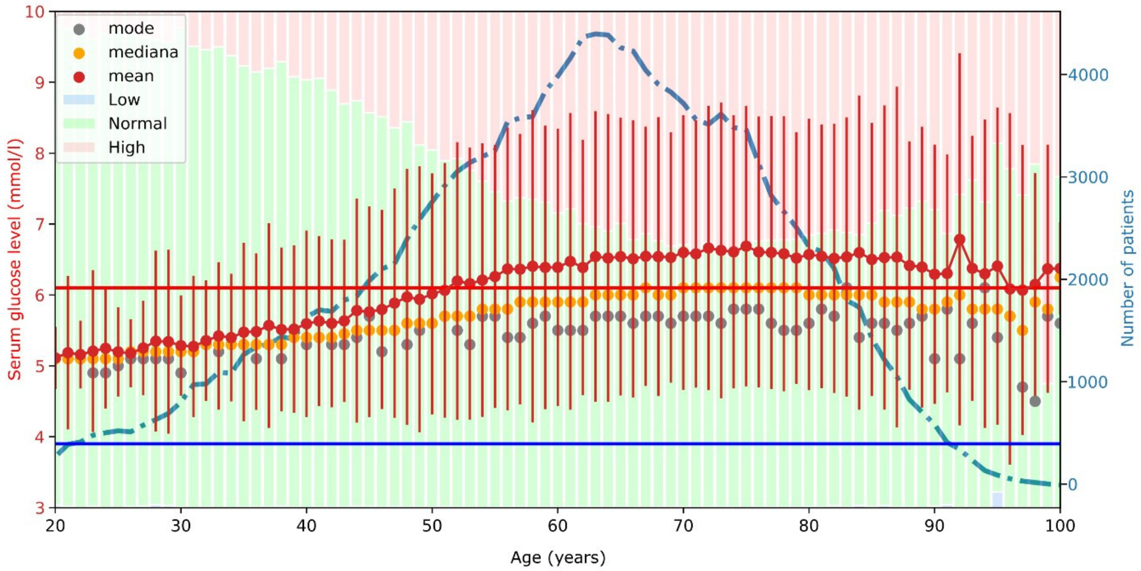

Figure S7: Age-specific blood glucose readings. The dashed blue line shows the absolute number of tests performed for a given age group. Gray, orange, and red circles indicate the mode, median, and mean blood glucose levels for a given age group. Vertical red lines indicate the standard deviation of blood glucose levels. Horizontal red line marks upper normal blood glucose level and horizontal blue line marks lower normal blood glucose level. Blue, green, and red bars mark the relative proportion of test results with low, normal, and high blood glucose levels, respectively.

The dynamics of serum glucose levels are gender-specific. The serum glucose levels in male and female patients in different age groups are given in Figure S8. In general, the distribution of blood glucose levels in any given age group is significantly different between males and females. Male patients have higher mean serum glucose levels and a higher dispersion in the same age group than female patients. The average serum glucose levels for male patients exceed the upped bond in the age groups 70-74 and 75-79. The mean serum glucose level for female patients never exceeds the upper bond. The share of male patients with above-normal serum glucose values increases faster than female patients and reaches its peak for the age group 70-75 year-old. The share of female patients with above-normal serum glucose levels is generally lower compared to male patients and reaches its peak value in the age group 75-79 year-old.

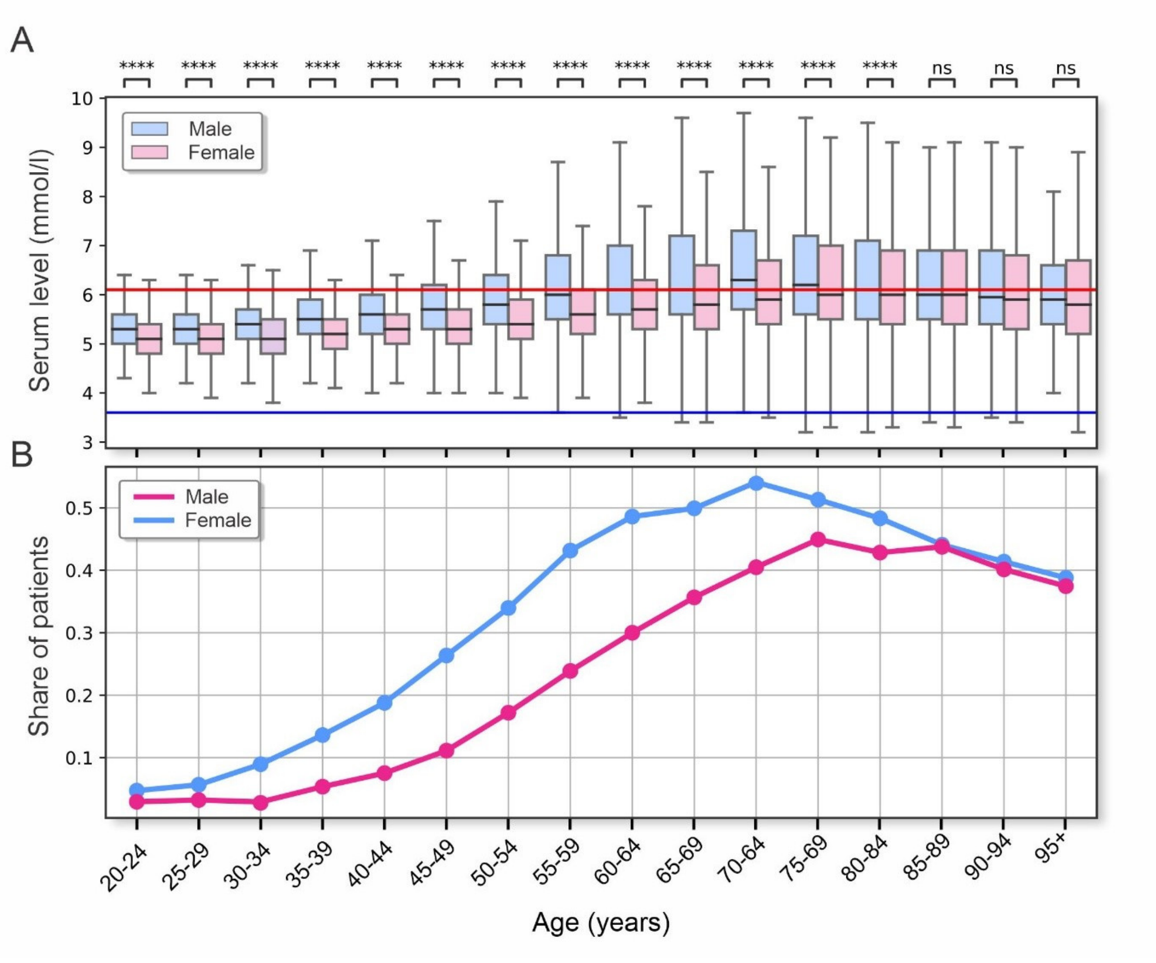

Figure S8: Age- and sex-specific serum glucose levels. (A) Distribution of serum glucose levels for different age groups and sexes. (B) Proportion of patients with elevated serum glucose levels for different age groups and sexes.

Statistical description of serum Gluc, Chol, LDL, HDL, and TG levels

Here we give an overview of all the laboratory tests considered. In Table S3, we report the percentage of patients who had abnormal test results. We analyzed male and female patients separately for each age group. To determine whether the percentage of abnormal tests was significantly different between male and female patients in a given age group, we used the chi-square test. If the corresponding p-value was less than 0.001, we rejected the null hypothesis, i.e., there is no difference between the percentage of abnormal test results between male and female patients.

Table S3: Proportion of patients, expressed as a percentage, who have abnormal serum glucose (Gluc), total cholesterol (Chol), LDL, HDL, and triglycerides (TG) in male and female patients for individual age groups and in the corresponding total population. Significant differences between proportions are highlighted with an asterisk indicating p-value < 0.001. For p-values > 0.001 we indicate the corresponding p-value. Statistical significance was calculated using the chi-square test.

	Age group								
Gluc	**20-29**	**30-39**	**40-49**	**50-59**	**60-69**	**70-79**	**80-89**	**90+**	**Total**
Male	3.7%	8.7%	17.9%	31.6%	44.9%	49.8%	44.3%	38.1%	34.6%
Female	1.9%	2.9%	7.0%	16.8%	31.3%	39.4%	37.3%	31.2%	23.8%
p-value	0.008	*	*	*	*	*	*	0.001	*
Chol									
Male	55.3%	65.1%	73.4%	77.0%	72.2%	66.9%	62.8%	60.8%	70.4%
Female	46.3%	55.3%	63.6%	71.0%	71.1%	63.8%	54.9%	48.4%	63.9%
p-value	*	*	*	*	*	*	*	*	*
LDL									
Male	47.8%	65.0%	74.6%	77.2%	71.6%	65.4%	62.2%	57.1%	70.0%
Female	38.9%	56.3%	6520.0%	71.2%	70.5%	6190.0%	53.4%	45.2%	63.4%
p-value	0.005	*	*	*	0.124	*	*	*	*
HDL									
Male	24.0%	37.2%	42.4%	45.6%	40.8%	33.3%	24.3%	20.0%	38.1%
Female	21.7%	28.1%	27.5%	30.6%	32.3%	29.3%	24.3%	23.3%	28.7%
p-value	0.376	*	*	*	*	*	0.977	0.134	*
TG									
Male	Male	24.0%	37.2%	42.4%	45.6%	40.8%	33.3%	24.3%	20.0%
Female	Female	21.7%	28.1%	27.5%	30.6%	32.3%	29.3%	24.3%	23.3%
p-value	p	0.376	*	*	*	*	*	0.977	0.134

We also compare the mean values of laboratory test results for male and female patients within a given age group. The results are presented in Table S4. Results are reported as mean ± 95% confidence interval. To determine whether the distribution of laboratory test results for a given test within an age group differed between male and female patients, we applied the Mann-Whitney U test. If the corresponding p-value was less than 0.001, we rejected the null hypothesis, i.e., there is no difference in the distribution of results in male and female patients.

Table S4: Mean ± 95% CI of serum glucose (Gluc), total cholesterol (Chol), LDL, HDL and triglycerides (TG) in male and female patients for individual age groups and in the corresponding total population. Significant differences between distributions are marked with an asterisk indicating p-value < 0.001. For pvalues > 0.001 we indicate the corresponding p-value. Statistical significance was calculated using the MannWhitney U test.

	Age group								
Gluc	**20-29**	**30-39**	**40-49**	**50-59**	**60-69**	**70-79**	**80-89**	**90+**	**Total**
Male	5.32 ± 0.06	5.52 ± 0.05	5.78 ± 0.04	6.15 ± 0.04	6.15 ± 0.04	6.69 ± 0.05	6.47 ± 0.07	6.34 ± 0.14	6.25 ± 0.02
Female 5	.09 ± 0.02 5.1	8 ± 0.02 5.35 ±	0.02 5.65 ± 0	.03 5.65 ± 0.0	6.31 ± 0.04 6	.46 ± 0.06 6.3	± 0.09 5.85 ±	0.02 p-value *	* * * * *
0.814 0.6	84 *								
Chol									
Male	4.49 ± 0.08	5.12 ± 0.05	5.49 ± 0.03	5.63 ± 0.03	5.37 ± 0.03	5.07 ± 0.03	4.87 ± 0.04	4.65 ± 0.09	5.29 ± 0.01
Female	4.70 ± 0.07	4.91 ± 0.04	5.16 ± 0.03	5.75 ± 0.02	5.92 ± 0.02	5.67 ± 0.03	5.36 ± 0.03	5.23 ± 0.06	5.54 ± 0.01
p-value	4.49 ± 0.08	5.12 ± 0.05	5.49 ± 0.03	5.63 ± 0.03	5.37 ± 0.03	5.07 ± 0.03	4.87 ± 0.04	4.65 ± 0.09	5.29 ± 0.01
LDL									
Male	2.72 ± 0.07	3.27 ± 0.04	3.53 ± 0.03	3.60 ± 0.03	3.37 ± 0.02	3.13 ± 0.03	3.01 ± 0.04	2.85 ± 0.08	3.33 ± 0.07
Female	2.70 ± 0.06	2.96 ± 0.03	3.21 ± 0.03	3.68 ± 0.02	3.78 ± 0.02	3.52 ± 0.02	3.27 ± 0.03	3.20 ± 0.06	3.47 ± 0.06
p-value	0.667	*	*	*	*	*	*	*	*
HDL									
Male	1.21 ± 0.03	1.19 ± 0.01	1.17 ± 0.01	1.19 ± 0.01	1.21 ± 0.01	1.21 ± 0.01	1.22 ± 0.01	1.21 ± 0.03	1.20 ± 0.03
Female	1.53 ± 0.03	1.48 ± 0.02	1.47 ± 0.01	1.48 ± 0.01	1.46 ± 0.01	1.46 ± 0.01	1.42 ± 0.01	1.35 ± 0.02	1.46 ± 0.03
p-value	*	*	*	*	*	*	*	*	*
TG									
Male	1.23 ± 0.08	1.52 ± 0.04	1.83 ± 0.05	1.91 ± 0.04	1.77 ± 0.03	1.59 ± 0.03	1.40 ± 0.03	1.26 ± 0.06	1.70 ± 0.01
Female	1.07 ± 0.04	1.07 ± 0.03	1.12 ± 0.02	1.35 ± 0.02	1.51 ± 0.02	1.50 ± 0.02	1.46 ± 0.02	1.45 ± 0.04	1.37 ± 0.01
p-value	*	*	*	*	*	*	*	*	*

Drug usage analysis

Drug usage of Insulin and analogs and lipid-modifying agents by age and gender is presented in Figure S9.

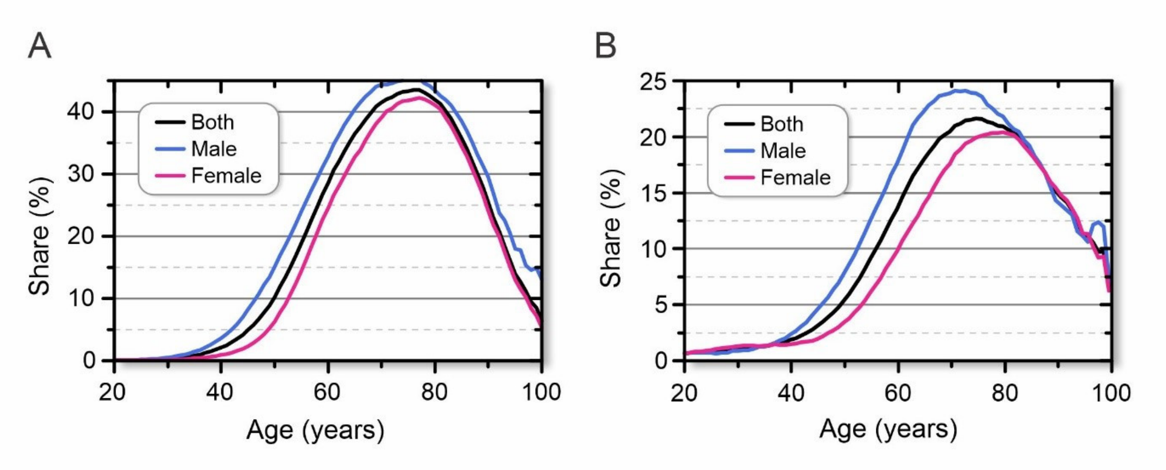

Figure S9: Age- and sex-specific drug use. (A) lipid-modifying agents and (B) anti-diabetic drugs. In both panels, the blue line is for men, the pink line is for women, and the black line is for both sexes.

The results shown in Figure S9 indicate that many more people are treated with drugs for lipid regulation than with drugs used for diabetes. Moreover, a certain proportion, about 15%, of the population is treated much earlier (about 10 years) with lipid regulation drugs than with diabetes drugs. In addition, the peak of medication use, which is reached around age 75, varies, reaching almost 45% of the corresponding population for dyslipidemia and only about 21% for hyperglycemia.

Diagnostic criteria for T2DM

As outlined in the American Diabetes Association's Standards of Medical Care in Diabetes (ADA), diabetes can be diagnosed based on plasma glucose criteria, including fasting plasma glucose (FPG) or 2-h plasma glucose (2-h PG) during a 75-g oral glucose tolerance test (OGTT), or HbA1c criteria [1]. More precisely, criteria for the diagnosis of diabetes as stated in [1] are:

- FPG ≥126 mg/dL (7.0 mmol/L).
- 2-h PG ≥200 mg/dL (11.1 mmol/L) during OGTT.
- HbA1c ≥6.5% (48 mmol/mol).
- In a patient with classic symptoms of hyperglycemia or hyperglycemic crisis, a random plasma glucose ≥200 mg/dL (11.1 mmol/L).

Each method has its advantages and disadvantages. The 2-h PG value diagnoses more people with prediabetes and diabetes. HbA1c assays may be used more generally to assess glycemic control in the clinic. However, it has some disadvantages, such as imperfect correlation between HbA1c value and average blood glucose in certain individuals.

Slovenian guidelines for the diagnosis of diabetes are comparable to the criteria of ADA. However, in Slovenia, diabetes is primarily diagnosed by random plasma glucose ≥ 11.1 mmol / l or FGP ≥ 7.0 mmol / l or plasma glucose 2 hours after the start of OGTT ≥ 11.1 mmol / l, the test is performed with 75 g glucose. The HbA1c test assay can be used as a diagnostic method for diabetes if all the necessary technical conditions are met and there are no conditions that would affect the accuracy of the measurement, or if the person is not suspected of having type 1 diabetes [2].

List of DFD and LMA drugs considered

In the drug database, each drug prescribed to a patient was assigned the appropriate anatomic therapeutic chemical (ATC) classification code, which is the accepted classification system for drugs maintained by the World Health Organization (WHO). In the present study, we focused on drugs classified as drugs for diabetes, DFD, (ATC A10) and lipid-modifying agents, LMA, (ATC C10). A complete list of all drugs considered under LMA and DFD with the corresponding prescription frequencies is provided in Tables S5 and S6, respectively. Prescribing frequency is calculated as the proportion of prescriptions for a given drug to the total number of prescriptions within a given drug class (i.e., LMA or DFD).

Table S5: List of LMA drug group with the corresponding ATC code and total frequency of prescriptions.

Rang	LMA drug name	ATC code	Frequency (%)
**1**	Rosuvastatin	C10AA07	40.8
**2**	Atorvastatin	C10AA05	33.1
**3**	Simvastatin	C10AA01	14.3
**4**	Fluvastatin	C10AA04	3.3
**5**	Fenofibrate	C10AB05	2.7
**6**	Pravastatin	C10AA03	2.3
**7**	Ezetimibe	C10AX09	1.5
**8**	Simvastatin and fenofibrate	C10BA04	0.7
**9**	Simvastatin and ezetimibe	C10BA02	0.5
**10**	Gemfibrozil	C10AB04	0.3
**11**	Rosuvastatin and amlodipine	C10BX09	0.2
**12**	Atorvastatin and amlodipine	C10BX03	0.1
**13**	Atorvastatin, amlodipine and perindopril	C10BX11	0.1
**14**	Lovastatin	C10AA02	0.1
**15**	Rosuvastatin and ezetimibe	C10BA06	0.0
**16**	Evolocumab	C10AX13	0.0
**17**	Alirocumab	C10AX14	0.0
**18**	Rosuvastatin and valsartan	C10BX10	0.0
**19**	Nicotinic acid, combinations	C10AD52	0.0

Table S6: List of DFD drug group with the corresponding ATC code and total frequency of prescriptions.

Rang	DFD drug name	ATC code	Frequency (%)
**1**	Metformin	A10BA02	35.7551
**2**	Gliclazide	A10BB09	15.4254
**3**	Insulin aspart	A10AD05	11.1608
**4**	Glimepiride	A10BB12	4.0453
**5**	Insulin (human)	A10AE01	3.7757
**6**	Insulin lispro	A10AD04	3.1567
**7**	Gliquidone	A10BB08	3.1481
**8**	Insulin glargine	A10AE04	2.7924
**9**	Metformin and sulfonylureas	A10BD02	2.3485
**10**	Glipizide	A10BB07	2.2022
**11**	Repaglinide	A10BX02	1.9791
**12**	Metformin and sitagliptin	A10BD07	1.7723
**13**	Insulin detemir	A10AE05	1.7473
**14**	Sitagliptin	A10BH01	1.4126
**15**	Metformin and vildagliptin	A10BD08	1.1050

**16**	Linagliptin	A10BH05	0.8944
**17**	Insulin glulisine	A10AB06	0.8431
**18**	Empagliflozin	A10BK03	0.8312
**19**	Acarbose	A10BF01	0.7846
**20**	Liraglutide	A10BJ02	0.7389
**21**	Glibenclamide	A10BB01	0.6106
**22**	Dapagliflozin	A10BK01	0.5642
**23**	Insulin degludec	A10AE06	0.4457
**24**	Dulaglutide	A10BJ05	0.4414
**25**	Metformin and empagliflozin	A10BD20	0.4014
**26**	Vildagliptin	A10BH02	0.3826
**27**	Metformin and dapagliflozin	A10BD15	0.3247
**28**	Exenatide	A10BJ01	0.2775
**29**	Insulin degludec and liraglutide	A10AE56	0.2775
**30**	Metformin and linagliptin	A10BD11	0.1659
**31**	Saxagliptin	A10BH03	0.1047
**32**	Lixisenatide	A10BJ03	0.0558
**33**	Insulin glargine and lixisenatide	A10AE54	0.0157
**34**	Metformin and saxagliptin	A10BD10	0.0124
**35**	Metformin and alogliptin	A10BD13	0.0005
**36**	Pioglitazone and alogliptin	A10BD09	0.0005
**37**	Alogliptin	A10BH04	0.0004

References:

1. Association, A.D. 2. Classification and Diagnosis of Diabetes: Standards of Medical Care in Diabetes—2020. *Diabetes Care* **2020**, *43*, S14–S31, doi:10.2337/dc20-S002.
2. Smernice za vodenje sladkorne bolezni (eng. Guidelines for diabetes management)., published by the Association of Endocrinologists of Slovenia. Accessible on: https://endodiab.si/wp-content/uploads/2015/12/3.-Diagnoza-SB.pdf
